# An Inverse Perspective Mapping-Based Approach for Generating Panoramic Images of Pipe Inner Surfaces

**DOI:** 10.3390/s23125363

**Published:** 2023-06-06

**Authors:** Sung Sic Yoo, Heung-Shik Lee

**Affiliations:** Department of Smart Mobility Engineering, Joongbu University, 305 Dongheon-ro, Deogyang-gu, Goyang-si 21713, Gyeonggi-do, Republic of Korea; sungsicyoo@gmail.com

**Keywords:** pipe inspection, inverse perspective mapping, image stitching, optical flow

## Abstract

We propose an algorithm for generating a panoramic image of a pipe’s inner surface based on inverse perspective mapping (IPM). The objective of this study is to generate a panoramic image of the entire inner surface of a pipe for efficient crack detection, without relying on high-performance capturing equipment. Frontal images taken while passing through the pipe were converted to images of the inner surface of the pipe using IPM. We derived a generalized IPM formula that considers the slope of the image plane to correct the image distortion caused by the tilt of the plane; this IPM formula was derived based on the vanishing point of the perspective image, which was detected using optical flow techniques. Finally, the multiple transformed images with overlapping areas were combined via image stitching to create a panoramic image of the inner pipe surface. To validate our proposed algorithm, we restored images of pipe inner surfaces using a 3D pipe model and used these images for crack detection. The resulting panoramic image of the internal pipe surface accurately demonstrated the positions and shapes of cracks, highlighting its potential utility for crack detection using visual inspection or image-processing techniques.

## 1. Introduction

Management of inner pipe walls is important because defects on the inner wall of the pipe can interfere with facility operations and pose a threat to safety. The main methods developed for crack detection in pipes include computer vision systems using cameras, magnetic field measurement, and acoustic detection using microphones [1]. With the advancement of robotics technology and artificial intelligence, computer vision-based pipe crack detection techniques have gained significant prominence. When applying computer vision-based crack detection techniques to pipes, two distinct challenges differentiate them from other engineering structures. The first challenge is the movement within the pipe itself. Since most pipes are located underground, accessing the interior is not easy. Many researchers have proposed methods for maneuvering pipe inspection robots to navigate pipes of various diameters and geometric structures [2,3,4]. The second challenge lies in obtaining images of the internal walls of pipes. Generally, the RVI (remote visual inspection) technique is employed, utilizing inspection robots or handheld videoscopes to explore the pipes and capture images of their internal walls [5,6,7]. To address the cost-saving and accuracy enhancement concerns associated with human-dependent visual inspections, various automated image-processing techniques, including artificial intelligence, are required [8,9,10]. For this purpose, surface images of structures, including cracks, are needed. However, unlike other engineering structures, the inner wall of a pipe is typically in the form of a long cylinder, making it difficult to obtain images of the entire surface. Furthermore, the images obtained while moving inside the pipe are perspective images, and the areas that need to be inspected for cracks are the surfaces of the pipe. Therefore, it is challenging to directly apply various automated image-processing techniques (including artificial intelligence developed for automated crack inspection) to the perspective images in order to facilitate automated crack inspection.

Various related studies have been conducted to obtain the surface images of pipes. For example, Pahwa et al. [11] devised a method using a 360-degree camera to capture continuous images inside the pipe and stitch them together. Kagami et al. [12] employed Structure-from-motion (SfM) techniques, utilizing 2D images to reconstruct 3D shapes and detect defects within the pipe. In [13], the authors estimated the relative positions of probes on the pipe and obtained undistorted images by combining a camera sensor with an integrated laser ring projector. Gunatilake et al. [14] proposed a mobile robot detection system that combines infrared laser profiling devices with stereo camera vision to generate a 3D RGB depth map, allowing for scanning, detecting, locating, and measuring internal defects in pipelines. In [15], the authors utilized an active stereo omnidirectional vision sensor to obtain panoramic images of the pipeline’s internal surface. Fang et al. [16] proposed an interesting method that restores the images of pipe walls using images captured from the front view. They addressed the radial distortion issue of the front-view images by employing nonlinear fitting features of a neural network and homemade feature stickers. These studies utilized high-resolution imaging equipment, such as 3D depth cameras that are capable of measuring the three-dimensional positional information of photographs or neural networks trained specifically for particular scenarios. However, these approaches have limitations in terms of their widespread usage due to the high cost involved in building high-performance equipment or the need for specialized artificial intelligence models.

Our proposed technique aims to generate images of the inner wall of a pipe by utilizing perspective images captured by a monocular camera sensor. Since the images of the pipe wall, captured while the sensor moves through the pipe, are perspective images projecting the wall from close to the camera to far away, we calculate the position of the corresponding wall on each pixel of the captured image. This calculation accounts for the focal length and the diameter of the pipe. The key unknown parameter that we need to determine is the depth on the pipe’s surface corresponding to a point on the image plane. The difficulty in finding this stems from the mapping between a two-dimensional plane and a three-dimensional curved surface. The problem becomes even more complex, particularly when the image plane is tilted and exhibits asymmetry. To obtain the relationship between a point on the image plane and a point on the corresponding wall when the pipe and the image plane are perpendicular, we first derive the inverse perspective mapping (IPM) formula for a cylinder. IPM is a transformation technique that can be used to map a 3D object projected onto a 2D plane to a new position to create a new 2D image. In [17,18,19], lane detection systems for autonomous driving, which is a well-known application of IPM, were employed. These systems utilize IPM to transform the perspective view of a road image into a top-view space, simplifying lane detection and eliminating perspective distortion from the resulting 2D image. We use the IPM to restore the sharpness of the image at an appropriate restoration distance, as the sharpness decreases with the increasing distance of the wall from the camera. Moreover, to correct the distortion that occurs due to obstacles during the movement of the robot inside the pipe, we derive a generalized formula that considers the angle of the image plane. This IPM formula is based on the position of the vanishing point in the perspective image. Another factor that contributes to the difficulty of this problem is the unknown position of the vanishing point. While a common method for vanishing point detection involves finding the intersection of the detected lines using the Hough transform [20], this method may not always apply to perspective pipe images due to the absence of a line component leading to the vanishing point. Therefore, we use optical flow [21,22] for vanishing point detection inside the pipe. Optical flow is a vector field that captures the motion of objects between consecutive frames by identifying the displacement of corresponding pixels in adjacent frames. To capture a panoramic image of the inner surface of a pipe, we first create a contour plot by using the vector magnitude of the optical flow formed in the perspective image of the pipe and detect circles. Next, we calculate the convergence positions of these circles to detect the vanishing point. We then connect the transformed images using image stitching techniques [23] and concatenate them into a panoramic image of the pipe wall. Ultimately, our technique generates a flat map of the pipe wall, which is convenient for crack detection. To validate our approach, we conduct numerical experiments using a 3D pipe model with arbitrary shape patterns and cracks inside.

## 2. Methods

In this section, we introduce an IPM-based method for obtaining a 2D image of the interior surface of a pipe from perspective images acquired while moving along the pipe. The principle of this method is to find a mapping that corresponds to cylindrical coordinates, derived from a polar coordinate system with the vanishing point as the origin, within the perspective image. The key unknown parameter we need to determine is the depth on the pipe that corresponds to a point on the image plane. In Section 2.1, assuming prior knowledge of the vanishing point, we derive an IPM-based formula to determine this depth. We first discuss the case of a perpendicular image plane and then derive the equation for the scenario where the image plane is tilted. Next, in Section 2.2, we present the image stitching technique that connects the transformed images to generate a panoramic image. Section 2.3 introduces the optical flow method to find the vanishing point. Finally, in Section 2.4, we provide an overview of the entire algorithm.

### 2.1. Inverse Perspective Mapping

#### 2.1.1. Perpendicular Image Plane

**Figure 1:**
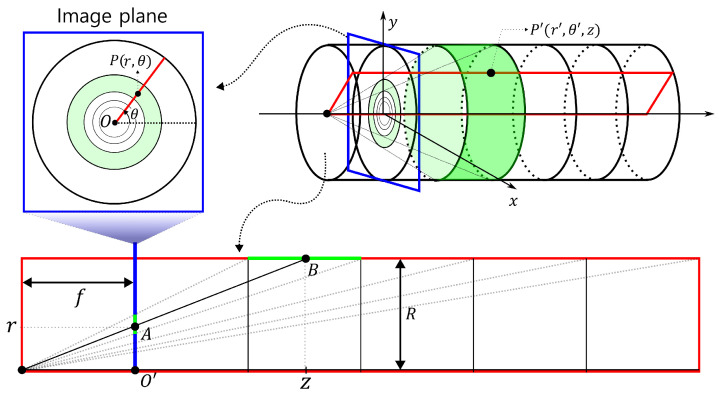
A perspective projection onto an image plane that is perpendicular to the piping.

Figure 1 illustrates the projection of the pipe’s interior surface onto the image plane within the pipe. We assume that the camera is moving through a straight pipe of a constant radius (*R*) and capturing perspective images. In Figure 1, the image plane, which is marked by a blue rectangle, is shown perpendicular to the direction of the flow inside the pipe. Suppose that circles are drawn at regular intervals inside the pipe, then the circles will be projected closer to the vanishing point as the camera moves further away from them. When representing the coordinates of the pipe wall in cylindrical coordinates and the image plane in polar coordinates, we can consider the following mapping between a point P(r,θ) on the image plane and a point $P^{\prime }\left(r^{\prime },\theta^{\prime },z\right)$ on the pipe wall:

(1) $$f:P\left(r,\theta \right)\to P^{\prime }\left(r^{\prime },\theta^{\prime },z\right)$$

where $\theta =\theta^{\prime }$ and $r^{\prime }=R$ (const.).

The red rectangle in Figure 1 represents a plane that shows the cross-section of the pipe for a fixed θ. Point *P* on the image plane corresponds to point *A* on the cross-section, while point $P^{\prime }$ on the pipe wall corresponds to point *B*. By a geometric proportion, we have the relationship r:R=f:f+z. Therefore, we obtain the following equation:

(2) $$z=f(\frac{R}{r}-1).$$

Here, *f* is the focal length, *R* is the radius of the pipe, and *r* is the distance from a point on the image plane to the vanishing point. Since *f* and *R* are both constants, we can observe that z+f and *r* are inversely proportional to each other, where z+f is the horizontal distance from the light source to the corresponding point on the pipe wall.

#### 2.1.2. Inclined Image Plane

**Figure 2:**
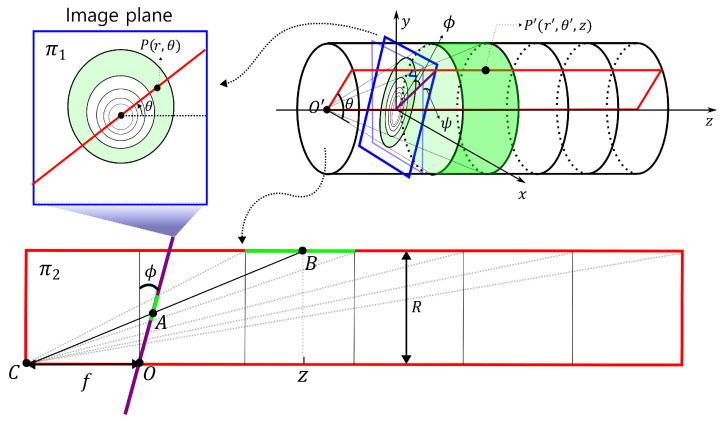
Perspective projection onto a tilted image plane.

Figure 2 shows the projection of the interior surface of the pipe onto the tilted image plane with an angle of ψ with respect to the x-axis. We define the rectangular region $\pi_{2}$ as the area where the interior of the pipe intersects the plane, which is created by rotating the half xz-plane with x>0 counterclockwise by an angle of θ. Let $l_{1}$ be the intersection line of $\pi_{2}$ and the image plane, $\pi_{1}$. The angle (ϕ) between $l_{1}$ and the *z*-axis is determined by θ and ψ, where ψ represents the angle at which the image plane rotates around the *x*-axis. Therefore, Equation (2) needs to be modified to include both θ and ψ as variables. As a result, point *P* on the image plane corresponds to point *A* on the cross-sectional plane, and the depth *z* of point *B* on the wall varies with the values of ψ and θ, which depend on the position of *A*.

**Figure 3:**
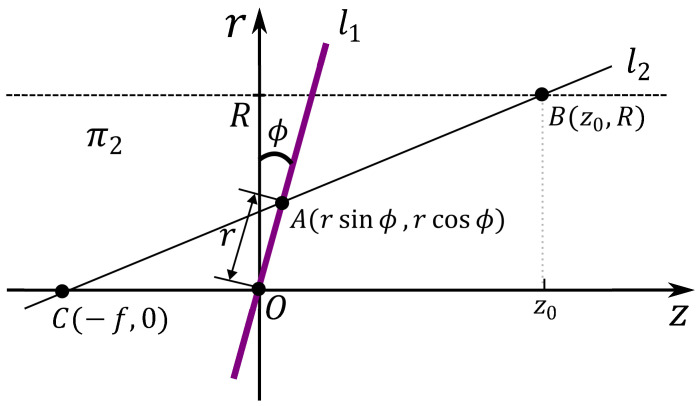
Correspondence between a tilted image plane and the interior wall surface of a pipe.

As shown in Figure 3, to determine the projection of a point (*A*) on the wall (*B*) from the image plane, we consider the line $l_{1}$ on the cross-sectional plane ($\pi_{2}$). Here, $l_{1}$ is the intersection of the image plane ($\pi_{1}$) and the cross-sectional plane ($\pi_{2}$), while *O* is the vanishing point on the image plane. Let *C* be the light source, then *A* is projected onto point *B* on the wall, and the line passing through these three points is denoted as $l_{2}$. The two lines are then expressed as follows:

(3) $$\begin{array}{cc} l_{1}: & r=\frac{z}{\tan\phi } \end{array}$$

(4) $$\begin{array}{cc} l_{2}: & r=\frac{R}{z_{0}+f}\left(z+f\right) \end{array}$$

Since point *A* lies on both $l_{1}$ and $l_{2}$, we obtain the following equation:

(5) $$\frac{R}{z_{0}+f}\left(r\sin\phi +f\right)=\frac{r\cos\phi }{\tan\phi }$$

Solving the equation for $z_{0}$ yields the following expression:

(6) $$z_{0}=R[\tan\phi +\frac{f}{r\cos\phi }]-f$$

Now, we aim to obtain a relationship equation for ϕ, which is determined by the rotation angle θ of the cross-sectional plane ($\pi_{2}$) and the tilt angle ψ of the image plane ($\pi_{1}$), as shown in Figure 2. Let $n_{1}$ and $n_{2}$ be the normal vectors to planes $\pi_{1}$ and $\pi_{2}$, respectively. We can obtain the rotated normal vectors by applying the rotations of ψ and θ to the original normal vectors, respectively, as follows:

(7) $$\begin{array}{cc} n_{1} & =\left[\begin{array}{ccc} 1 & 0 & 0\\ 0 & \cos\psi & -\sin\psi \\ 0 & \sin\psi & \cos\psi \end{array}\right]\left[\begin{array}{c} 0\\ 0\\ 1 \end{array}\right]=\left[\begin{array}{c} 0\\ -\sin\psi \\ \cos\psi \end{array}\right], \end{array}$$

(8) $$\begin{array}{cc} n_{2} & =\left[\begin{array}{ccc} \cos\theta & -\sin\theta & 0\\ \sin\theta & \cos\theta & 0\\ 0 & 0 & 1 \end{array}\right]\left[\begin{array}{c} 0\\ 1\\ 0 \end{array}\right]=\left[\begin{array}{c} -\sin\theta \\ \cos\psi \\ 0 \end{array}\right]. \end{array}$$

The direction vector *u* of $l_{1}$ is the cross product of $n_{1}$ and $n_{2}$, which can be expressed as follows:

(9) $$u=n_{1}\times n_{2}=\left[\begin{array}{c} \cos\theta \cos\psi \\ \sin\theta \cos\psi \\ \sin\theta \sin\psi \end{array}\right]$$

Now, considering the relationship between u and the direction vector $e_{3}$ of the *z*-axis, we have:

(10) $$\cos\left(\frac{\pi }{2}-\phi \right)=\sin\phi =\frac{u\cdot e_{3}}{\left\|u\right\|}=\frac{\sin\theta \sin\psi }{\sqrt{\cos^{2}\psi +\sin^{2}\theta \sin^{2}\psi }}$$

As a result, we obtain the following equation, which determines the depth of corresponding points on the cylindrical surface for the tilted image plane:

(11) $$\begin{array}{cc} z & =R[\tan\phi +\frac{f}{r\cos\phi }]-f \end{array}$$

(12) $$\begin{array}{cc} \phi & =\sin^{-1}(\frac{\sin\theta \sin\psi }{\sqrt{\cos^{2}\psi +\sin^{2}\theta \sin^{2}\psi }}) \end{array}$$

Here, *f* is the focal length, *R* is the radius of the pipe, and *r* is the length of $\bar{OA}$. Note that when ψ=0, θ=0, or $\frac{\pi }{2}$, Equation (11) reduces to the same as Equation (2).

### 2.2. Image Stitching

To generate a complete interior wall image of the pipe by connecting the images converted to a 2D wall, we use the panoramic image stitching technique [23,24]. This image stitching technique finds common features among multiple images taken of the same location or object and connects them into a single image. We use the SIFT (scale-invariant feature transform) method to identify corresponding points between two images, analyze the geometric relationship between the image pairs, and establish the transformation necessary to align and convert one image to another. Considering that the resolution of the images captured inside the pipe decreases with increasing observation depth, we carefully select and crop specific regions of the captured images for the purpose of image stitching, with the aim of obtaining optimal results despite the decreasing resolution.

### 2.3. Vanishing Point Detection

**Figure 4:**
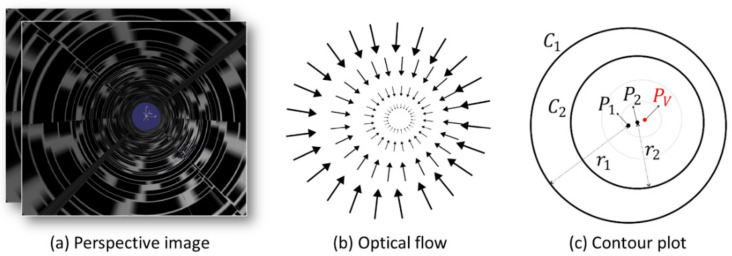
Detection of vanishing points using the optical flow from successive perspective images of the interior wall surfaces of pipes: (**a**) perspective images, (**b**) optical flow, (**c**) contour plot representing the levels of the optical flow vector magnitude.

We detect the vanishing point using the optical flow obtained from a sequence of perspective images. Figure 4 shows a conceptual diagram that explains the method of detecting the vanishing point. Optical flow is calculated for two consecutive perspective images obtained during movement inside the pipe, as shown in Figure 4a. In order to address the aperture problem, we employed the Gunnar–Farneback optical flow method using the Python OpenCV library. As can be seen in Figure 4b, each vector in the optical flow has a direction toward the vanishing point, and the size decreases as it approaches the vanishing point. Theoretically, the maximum intersection point of the optical flow vectors is the optimal position for estimating the vanishing point. However, in practice, this method’s accuracy is often limited by minor errors in the vectors, which can lead to significant positional deviations in the vanishing point estimation. Therefore, we instead generate a contour plot for the magnitudes of the optical flow vectors, as shown in Figure 4c. Circles are generated along the pipe wall in this contour plot. We select two circles among the circles generated around the vanishing point. Various methods for automatically detecting circles in images have been studied [25,26], but in the present study, we manually select three points on each of the two contours we want to choose on the contour plot to detect the circles. For the two detected circles, $C_{1}$ and $C_{2}$, with respective centers, $P_{1}$ and $P_{2}$, and respective radii, $r_{1}$ and $r_{2}$ (where $r_{1}>r_{2}$), we use an infinite geometric series to calculate the position of the vanishing point $P_{v}$ as follows:

(13) $$P_{v}=P_{1}+\frac{1}{1-r}\left(P_{2}-P_{1}\right),$$

where $r=r_{2}/r_{1}$.

### 2.4. Algorithm

**Figure 5:**
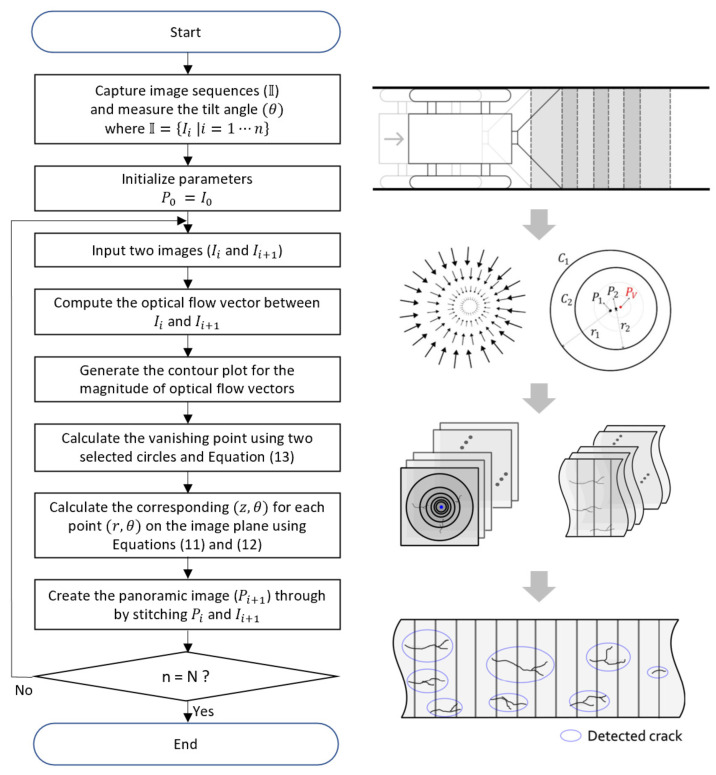
An overall algorithm for generating a panoramic image of the interior wall of a pipe based on inverse perspective mapping.

Figure 5 represents the overall algorithm that we propose for creating a panoramic image of the inside wall of a pipe. It can be summarized into the following four-step process:

- STEP 1. Capture the image sequences of the inside of the pipe while moving through the pipe, and measure the tilt angles (θ).
- STEP 2. Compute the optical flow for the consecutive images and generate a contour plot for the optical flow magnitudes. Select two circles from the contour plot and determine the vanishing point location using Equation (13).
- STEP 3. Obtain images of the pipe wall corresponding to each perspective image for the collected image sequence using Equations (11) and (12).
- STEP 4. Stitch the transformed images together using image stitching to create a panoramic image and detect cracks.

## 3. Results

To validate our method, we generated a sufficiently long 3D pipe using CATIA and inserted images with irregular patterns and cracks on the inner wall of the pipe. The diameter of the pipe was 350 mm and the focal length was 420 mm. We extracted a total of 50 perspective images by moving 40 mm inside the pipe while capturing images. We considered cases where the shooting angle was perpendicular to the direction of the pipe and cases where the pitch angle was tilted by 0.1 degrees.

### 3.1. Vanishing Point Detection

**Figure 6:**
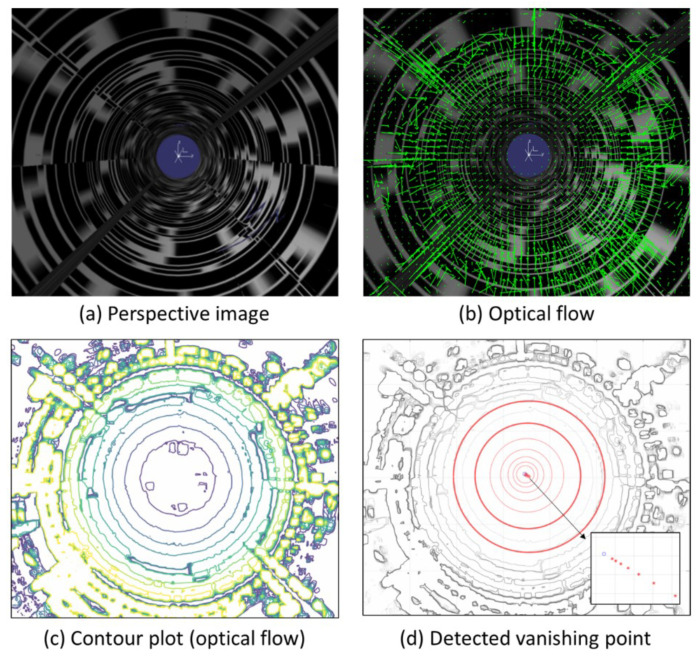
Vanishing point detection using optical flow.

Figure 6 illustrates the process used to detect the vanishing point using the optical flow for a sequence of perspective images obtained by traversing inside the pipe model. The optical flow is calculated using the perspective image sequence shown in Figure 6a, and the green arrows in Figure 6b represent the optical flow (note that the white dot in the center here is not the vanishing point but simply the coordinate axis). As the vectors move closer to the center, their sizes decrease, while their magnitudes increase as they move outward, with errors in the direction. Figure 6c shows a contour plot of the magnitude of the optical flow vectors. Several circles can be seen near the vanishing point. The two circles selected from the contour plot, which are indicated by thick red lines in Figure 6d, were used to create circles by selecting three points for each circle on the contour plot. In Figure 6d, the two circles marked with bold red lines are selected from the circles observed in the contour plot. We generated the circles on the contour plot by selecting three points for each of the two circles. The red circles within the selected circles are drawn based on estimates of the centers and radii of the two selected circles. We can see that the centers of the circles converge to the blue vanishing point in the magnified image at the bottom right of Figure 6d.

### 3.2. Inverse Perspective Mapping

#### 3.2.1. Perpendicular Image Plane

**Figure 7:**
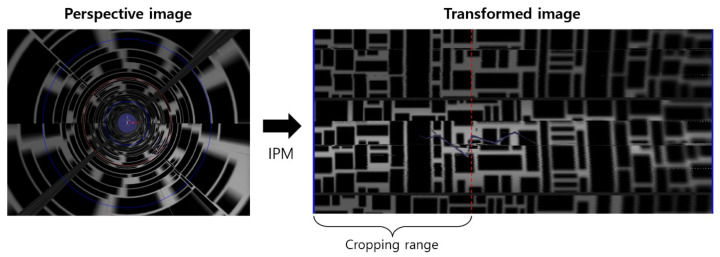
Generation of wall images using IPM when the flow direction of the image plane is perpendicular to the pipe. The red dashed lines indicate the cropping range.

**Figure 8:**
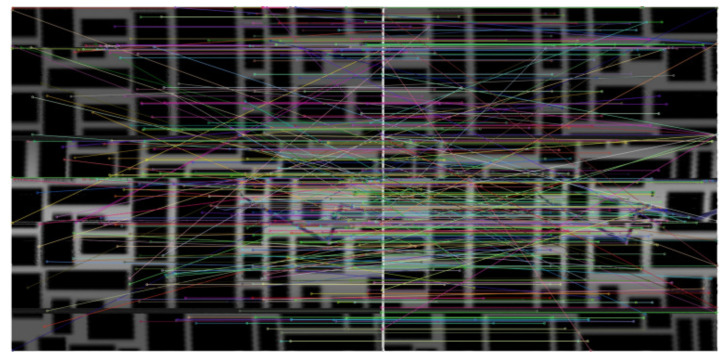
Image registration between two IPM-transformed images.

**Figure 9:**
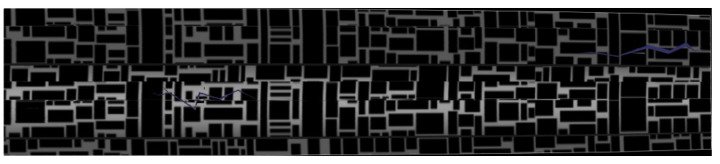
Panoramic image of the pipe wall created by connecting IPM-transformed images using image stitching.

The left side of Figure 7 shows a perspective image when the image plane is perpendicular to the direction of the pipe. Two circles (blue line) with radii $r_{1}$ and $r_{2}$ $\left(r_{1}>r_{2}\right)$ were generated based on the vanishing point to set the area to be transformed. The right image in Figure 7 shows the resulting image plane that has been transformed using Equation (2). We used the scatter function in MATLAB to pass RGB values to corresponding positions. As can be seen in the transformed image in Figure 7, as we move toward the right, deeper areas of the pipe are represented, thus resulting in decreased resolution and slight image distortion. Therefore, in this experiment, we set the cropping area to points satisfying $\frac{4}{7}r_{1}<r<r_{1}$, where *r* is the distance from the vanishing point to the image plane. The transformed images are connected using the stitching technique described in Section 2.2. For image stitching, we adopted the OpenCV library in Python. Figure 8 shows feature matching for two sequential images obtained using the SIFT algorithm. Figure 9 shows the results from connecting the transformed images using image stitching, thus enabling the easy identification of crack positions inside the pipe.

#### 3.2.2. Inclined Image Plane

**Figure 10:**
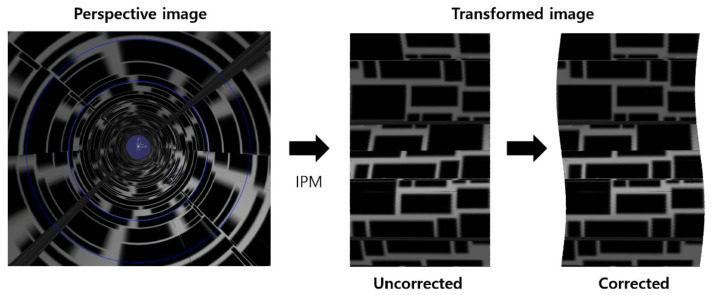
Image correction for the case when the image plane is tilted.

Figure 10 on the left shows a perspective image obtained when the image plane is tilted by 0.1 degrees in pitch. The blue circle represents the cropping range. If we use Equation (2) without considering the tilt of the image plane, the resulting image is distorted, as shown in the middle image in Figure 10. However, when we use Equations (11) and (12) to consider the tilt of the image plane, the transformed image no longer has a rectangular boundary and instead has a wavy shape, and the image distortion disappears. To perform actual image stitching, we set the cropping range to exclude the wavy area and accordingly calculated the internal square.

## 4. Discussion

In our paper, we propose a technique for generating the panoramic image of the in-pipe surface by transforming perspective images obtained from inside the pipe. Firstly, we applied the optical flow technique to consecutive perspective images, generating contours and calculating the converging positions of circles to detect the vanishing point. The obtained vanishing point was used as the reference to rotate cross-sections and derive the positions of inverse projection points on the image plane. To correct the image distortions caused by the tilting of the image plane, we derived an IPM-based formulation by considering the intersection of the image plane and the cross-sectional plane, based on the tilting angle.

Through numerical experiments, we confirmed that perspective images obtained from a 3D pipe model could be restored to images of the pipe wall using IPM. The computational time for image transformation using the derived IPM-based formulation was efficient, taking approximately 0.16 s per image, without the need for matrix inversions. We also observed the effective distortion correction of the image even when the image plane was tilted. The manually calculated vanishing point based on passive circle extraction showed reasonable estimations but still exhibited limitations dependent on circle detection accuracy. As a result, the obtained panoramic image of the internal pipe surface accurately displayed the positions and shapes of cracks, indicating its potential usefulness for crack detection based on visual inspection or imaging processing techniques.

## 5. Conclusions

This paper aims to develop a cost-effective and simple algorithm for generating complete images of the inner walls of pipes. Our contributions are as follows:

- We developed a simple algorithm based on inverse perspective mapping (IPM) that generates images of the entire pipe wall using only image sequences obtained from a monocular camera, without the need for high-performance capturing equipment.
- We proposed a new technique based on optical flow for obtaining vanishing points in perspective images of the pipe interior with few line edges.
- We presented formulas to address image distortion issues caused by shooting angles when applying IPM.
- We proposed a new technique that utilizes optical flow in perspective images of the pipe interior, in cases with limited line edges, to obtain the vanishing point.
- Our algorithm enables the acquisition of an end-to-end image of the pipe through image stitching, allowing for the detection of crack severity and location.
- Furthermore, we can obtain a 360-degree image of the pipe interior without the need for camera rotation.

Our limitations and future research topics are as follows: In this paper, we did not propose a method for estimating the tilted angle of the image plane. Additional considerations should be given to methods for calculating the tilt of the image plane, such as using inertial measurement unit (IMU) sensors or directly measuring the tilt. The automated extraction of circles using the circle Hough transform in the vanishing point detection process requires further research. We expect these issues to be further developed and experimentally validated through future research.

## Data Availability

Data are available from the authors upon reasonable request.

## Abbreviations

The following abbreviations are used in this manuscript:

IPM	inverse perspective mapping
RVI	remote visual inspection
IMU	inertial measurement unit
SIFT	scale-invariant feature transform
